# The Social Worker in the United States

**Published:** 1921-02-26

**Authors:** 


### The Social Worker in the United States.

In a recent number of *Maternity and Child Welfare* Miss Hilda Cashmore discusses the term "social work" and the attempt made in this country in the last two years to organise social workers into a professional union. In the United States the term has a recognised interpretation; there medical social service in hospitals has passed the formative stage and is accepted as a distinct department of the hospital. It has its own organisation in the American Association of Hospital Social Workers, and its own journal, entitled *Hospital Social Service*, which after two years of publication as a quarterly has with the New Year become a monthly magazine. The first monthly issue contains the survey of hospital social work in the United States made by the American Hospital Association last year; an account of social work in hospitals of Toronto, by Mr. Robert Mills, of the Toronto Health Department; an article by O. M. Lewis and two collaborators of the Division of Venereal Disease of the Massachusetts General Hospital; and a discussion of methods of Parental Authority, by Miss J. L. Beard. Besides news notes and abstracts of articles of interest to the medical social worker, there are departments devoted to the American Association of Hospital Social Workers, and to cardiac, nutritional, and handicap work. We wish the new monthly every success.

